# Lip closing force of Class III patients with mandibular prognathism: a case control study

**DOI:** 10.1186/1746-160X-10-33

**Published:** 2014-08-26

**Authors:** Sihui Chen, Ying Cai, Fengshan Chen

**Affiliations:** 1 Department of Orthodontics, Laboratory of Oral Biomedical Science and Translational Medicine, School of Stomatology, Tongji University, Middle Yanchang Road 399, Shanghai, P. R. China

**Keywords:** Lip closing force, Mandibular prognathism, Class III, Perioral force

## Abstract

**Introduction:**

To compare the lip closing force of patients with mandibular prognathism to that of patients without dentofacial anomalies.

**Methods:**

The subject group included 62 female patients of Class III relationship with mandibular prognathism. The control group been comprised of 71 patients of Class I relationships without skeletal deformities. Maximum lip closing force and average lip closing force were measured using a Y-meter. Student’s *t*-test was carried out to analyse the differences between the groups. Correlation and stepwise multiple linear regression analyses were performed to analyse the relationship between lip closing force and craniofacial morphology.

**Results:**

The lower lip closing force of subjects with mandibular prognathism was significantly greater than that of patients in the control group (P < 0.001), while the upper lip closing force showed no difference (P > 0.05). The lower lip closing force of patients with mandibular prognathism was strongly correlated with IMPA (Lower Incisor - Mandibular Plane angle, P < 0.001) and FMA (Frankfort Plane-Mandibular Plane angle, P < 0.001). Multiple regression equations: (MaxLL) = 12.192 - 0.125 * (IMPA) + 0.082 (FMA); (AveLL) = 9.112 - 0.091 * (IMPA) + 0.054 (FMA).

**Conclusions:**

The lower lip closing force was markedly increased in Class III patients with mandibular prognathism and was strongly correlated with lower incisor position and mandibular plane angle.

## Introduction

High prevalence of Class III malocclusions has been found in Asian populations. Kitai [1] reported that 5-20% of the Japanese population possessed the characteristics of Class III malocclusion. Similarly, Johnson [2] discovered a prevalence of 23% in Chinese children.

Studies have indicated that 63–73% of Class III malocclusions are of the skeletal type [3], while in the research of Mackay [4], those patients who required surgical correction of Class III conditions all had some degree of mandibular prognathism, which has long been viewed as one of the most severe maxillofacial deformities [5]. Indeed, mandibular prognathism, which is commonly related to Class III malocclusion, is a facial disharmony for which patients frequently seek treatments [6].

However, the etiological mechanisms of this condition still remain unrevealed. Class III malocclusion is not a distinct clinical entity, and it can exist in with any number of combinations of skeletal and dental components. Among the possibilities, muscular factors could constitute a vital component, based on commonly held perspectives.

Complex interdependence exists among teeth, perioral force and jaws. Ideal dentition arises from an equilibrate system composed of intraoral forces, represented by the tongue, and extraoral forces, represented by the lip force. If the force balance collapses, for example the lip force is less powerful than a normal condition, teeth inclination would change towards the weaker side. More complicated mechanism may be involved, but similar force system is very important to the growth and development of the dentoalveolar morphology. These soft tissue matrices, and particularly labial pressure from the circumoral musculature, may influence the outcome of craniofacial growth [7]. For example, hypofunction of mentalis muscle, resulting in less restriction on mandibular growth, appears to be related to mandibular protrusion [8]. A sound understanding of the surrounding soft tissues and their biological behaviour, especially labial pressure, which is represented by lip closing force [4], could help to reveal the etiology of dentoalveolar dysplasia.

Concerning the relationship between lip-closing force and craniofacial morphology, the closing force of the upper lip has a great influence on maxillary incisor angulation, vertical skeletal pattern and lip protrusion [9,10]. As to the skeletal pattern, most of the previous researches have focused on both Class I and Class II malocclusions [9,11-15]. Hardly any research has concentrated on Class III malocclusions, except Ueki [16], who demonstrated that the LCF of postoperative Class III patients were higher than the preoperative status. After all, the related data remain incomplete.

Our research group restricted our subjects to those with simplex mandibular prognathism to eliminate confounding factors, such as maxillary retrognathism and asymmetry, so that we could define a possible mechanism regarding how LCF influences the ultimate outcomes of craniofacial growth in Class III conditions.

The purposes of this study were to define both the upper and lower LCFs acting in Class III malocclusion patients with mandibular prognathism and to indicate the possible relationships between LCF and craniofacial morphology.

## Materials and methods

**Figure 1:**
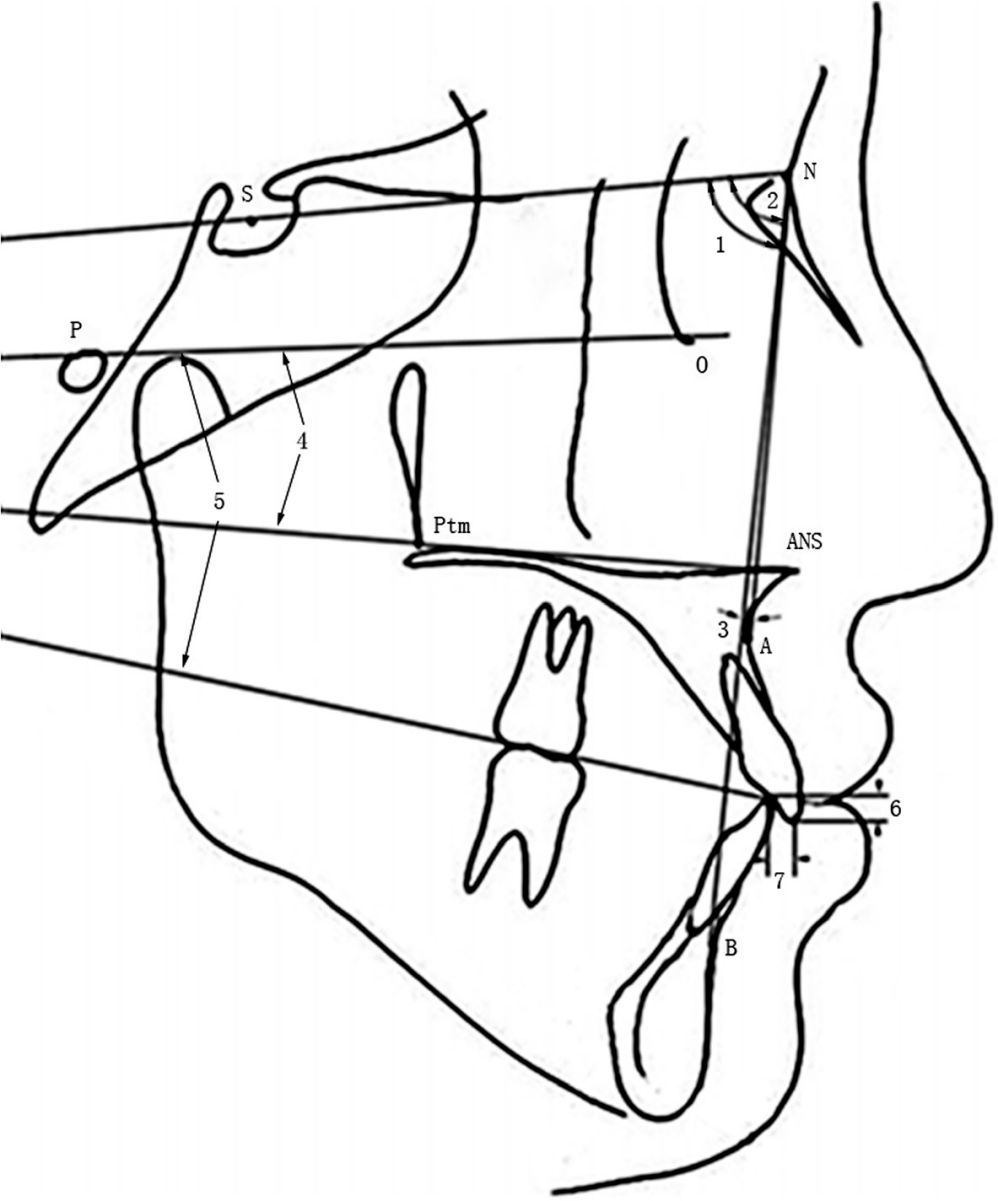
**Measurements used in this study.** 1, SNA (Sella–Nasion-A Angle); 2, SNB (Sella-Nasion-B Angle); 3, ANB (A-Nasion-B Angle); 4, PP-FH (Palatal Plane - Frankfort Plane Angle); 5, OP-FH (Occlusal Plane - Frankfort Plane Angle); 6, overbite; 7, overjet.

**Figure 2:**
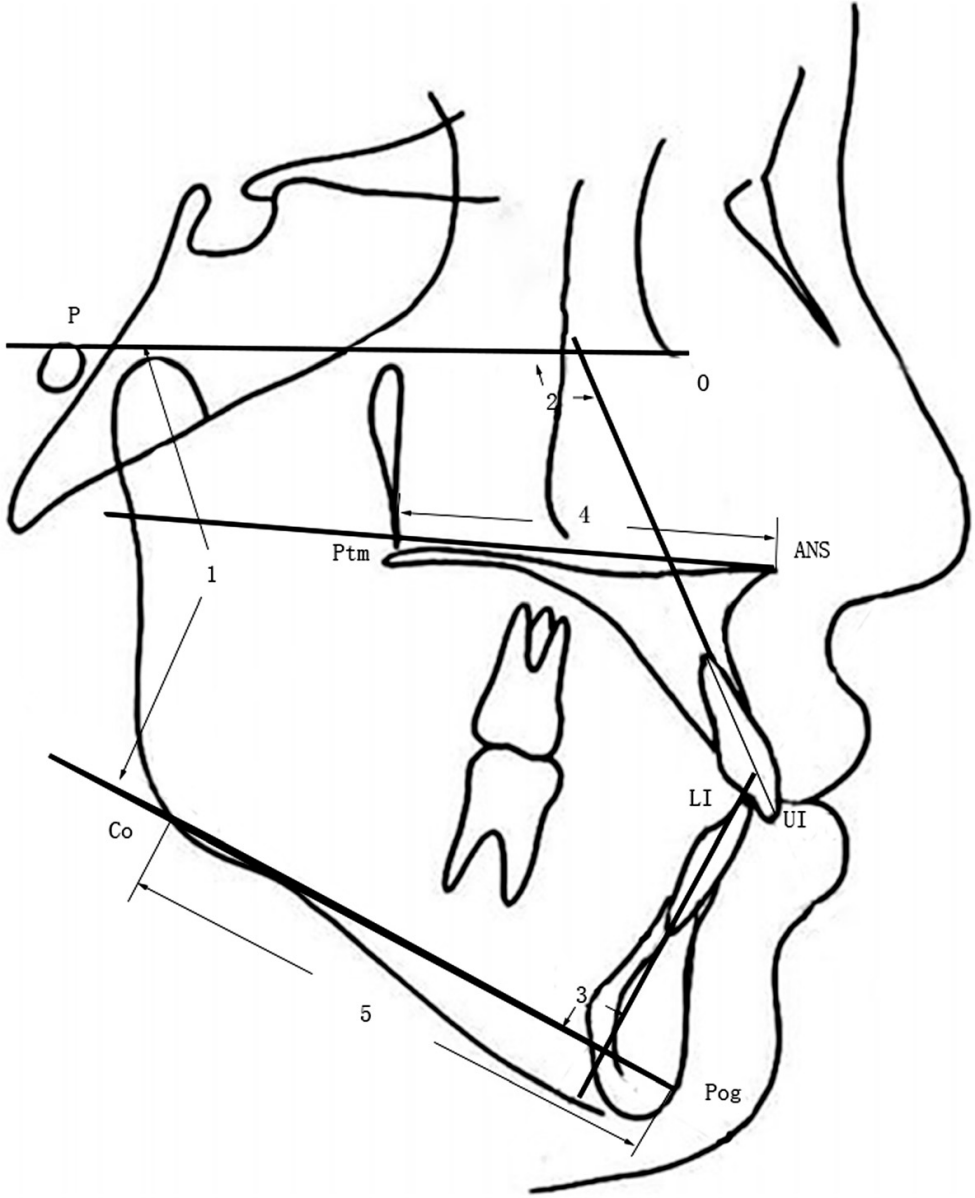
**Measurements used in this study (continued).** 1, FMA (Frankfort Plane - Mandibular Plane Angle); 2, U1-FH (Upper Incisor - Frankfort Plane Angle); 3, IPMA (Lower Incisor - Mandibular Plane Angle); 4. ANS-Ptm (length of maxilla); 5, Co-Pog (length of mandible).

All the patients who took part in this study were newly diagnosed patients who requested orthodontic treatment over a three-year period in the Orthodontic Department of Tongji Stomatological Hospital. The subject group (Group 1) consisted of 62 female patients (average age 17.92 ± 1.65 y) with Class III malocclusion. All the cases were diagnosed as skeletal Class III, characterized by mandibular prognathism but with relatively normal positioning of the maxilla on the basis of lateral cephalographic analysis. The control group (Group 2) consisted of 71 female patients (average age 18.20 ± 1.46 y) in our department with Class I skeletal patterns and Class I occlusal relationships without skeletal deformity. The inclusion criteria were: no loss of permanent teeth; no missing or supernumerary teeth; no history of orthognathic surgery or previous orthodontic treatment; no congenital craniofacial anomalies; and no occlusal canting or other asymmetric skeletal patterns.To evaluate the occlusal and skeletal patterns of each subject, cephalograms were obtained. The measurement landmarks were SNA (Sella–Nasion-A Angle, represent maxilla position toward cranium), SNB (Sella-Nasion-B Angle, represent mandible position toward cranium), ANB (A-Nasion-B Angle, represent mandible position toward maxilla), PP-FH (Palatal Plane - Frankfort Plane Angle), OP-FH (Occlusal Plane - Frankfort Plane Angle), overbite, overjet, UI-FH (Upper Incisor - Frankfort Plane Angle), FMA (Frankfort Plane - Mandibular Plane Angle), IMPA (Lower Incisor - Mandibular Plane Angle), ANS-Ptm (length of maxilla) and Co-Pog (length of mandible). All the landmarks (Figure 1 and Figure 2) were identified and digitized by the same investigator, utilizing Dolphin Imaging software (version 10.5, Dolphin, Imaging and Management Solutions, Chatsworth, CA, USA).

**Figure 3:**
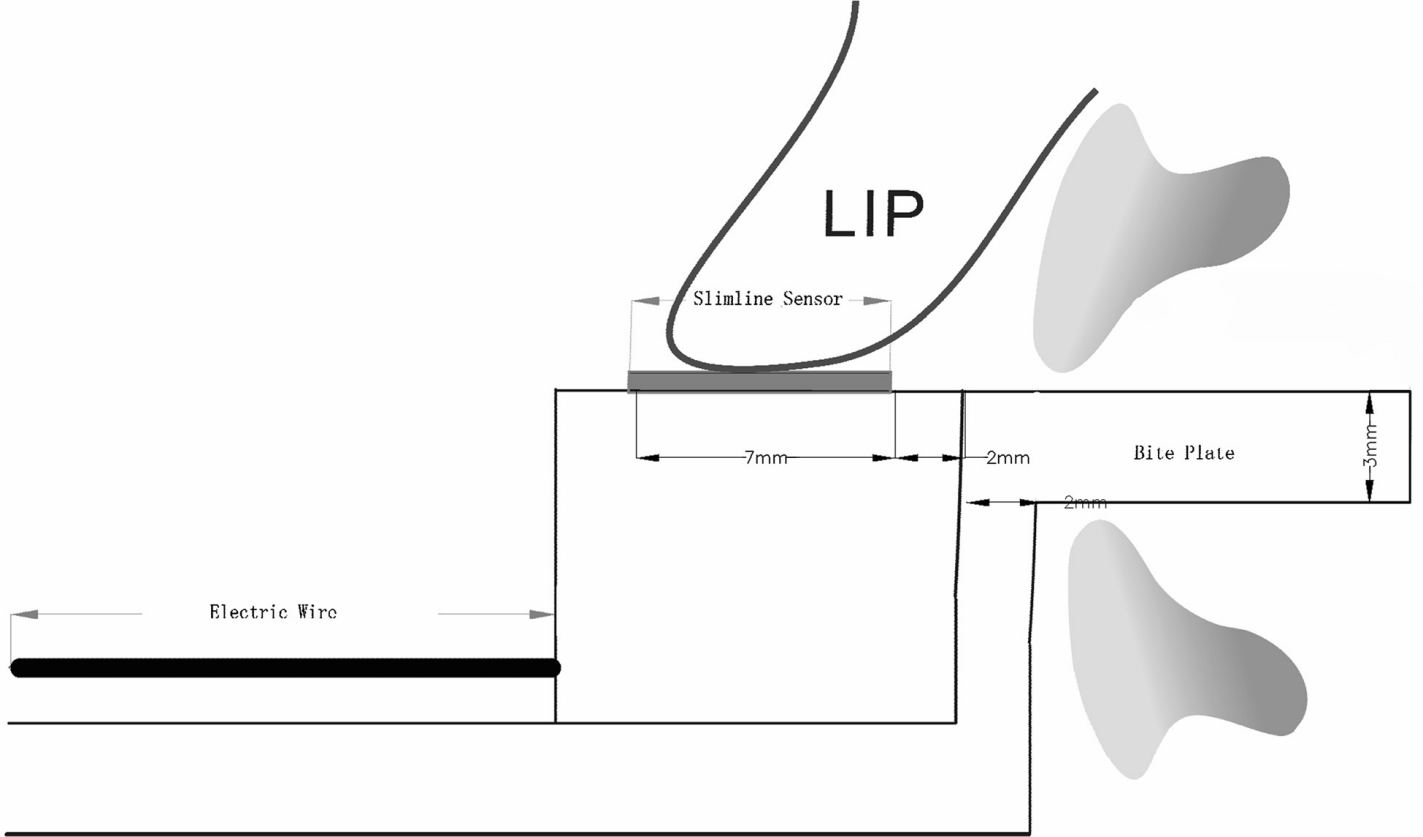
Lateral view of the Y-meter schematic diagram.

The Y-meter, the instrument for LCF measurement, has previously been used in other studies [9,10] (Figure 3). The biteplate was coated with baseplate wax before each measurement to help the subjects to hold with their incisors in position. The Slimline Sensor (9131A49, Kistler Co. Winterthur, Switzerland) was located on the upper surface of the horizontal plate of the Y-meter, with which the lip under measurement status maintained contact. The quartz sensor was intended to measure dynamic and quasi-static forces. Its characteristics, high resolution, high rigidity and extremely small dimensions render it ideal for measuring the vertical vector of the lip closing force. A base charge amplifier (5034A10, Kistler Co.) and DASYLAB (DASYTEC, Amherst, NH, USA) software, version 5.50, were also applied for data acquisition. The Y-meter was stored at 37°C to minimize temperature-induced errors.

After a brief explanation was provided and several practice measurements were taken, each subject was requested to close his or her lips as tightly as possible. The LCF was monitored for 5 seconds. The maximum value and the average value for 5 seconds were measured with the DASYLAB software. Measurements were generated three times at 3-minute intervals. After measuring the upper LCF, the identical procedure was used to evaluate the closing force of the lower lip with the Y-meter upside down. We recorded the LCF values with the patients in the natural head position.

Student’s *t*-test was carried out to analyse the differences between the groups. Pearson’s product moment correlation test was used to analyse the relationship between lip force and craniofacial morphology. Stepwise multiple linear regression analysis, with the average and maximum values of both LCFs, was further performed. All the statistical analyses were performed with SPSS software (SPSS Inc., Chicago, IL, USA), version 20 for Windows.

To evaluate the magnitude of measurement error involved in this study, lateral cephalograms of 12 randomly selected subjects were retraced, redigitized and reanalyzed after a 2-week interval, and the error was calculated by Dahlberg’s formula [17]. The error ranged between 0.04 mm and 0.20 mm for the linear measurements and between 0.15° and 0.90° for the angular measurements. All the measurement procedures and cephalometric analyses were performed by the same researcher.

This study took the Declaration of Helsinki on medical protocol and ethics and the regional Ethical Review Board of Tongji University approved the study. The researcher previously obtained the informed consent of all the subjects. The rights of our subjects were protected, and the data were only for research use.

## Results

The means and standard deviations for each variable are shown in Table 1. All the cases in the subject group were diagnosed as skeletal Class III, characterized by mandibular prognathism (average ANB -3.67 ± 0.86, SNB 84.63 ± 1.16) and normal positioning of the maxilla (average SNA 80.96 ± 1.37). No skeletal deformities existed among the patients in the control group (average SNA 81.98 ± 1.23, SNB 80.87 ± 1.29 and ANB 1.11 ± 0.79).

**Table 1:** Mean and SD of each variable

	**Group 1**	**Group 2**
SNA	80.96 ± 1.37	81.98 ± 1.23
SNB	84.63 ± 1.16	80.87 ± 1.29
ANB	-3.67 ± 0.86	1.11 ± 0.79
PP-FH	2.35 ± 0.72	1.95 ± 0.47
OP-FH	11.93 ± 1.19	12.18 ± 1.23
overbite	1.86 ± 1.05	2.65 ± 0.77
overjet	-3.24 ± 0.84	2.52 ± 0.79
UI-FH	74.07 ± 2.25	71.71 ± 2.17
FMA	34.43 ± 3.03	30.55 ± 2.56
IMPA	78.54 ± 3.88	87.77 ± 4.58
Ans-Ptm	46.77 ± 1.90	47.05 ± 1.87
MaxUL	7.59 ± 1.00	7.63 ± 0.67
AVeUL	5.94 ± 1.25	6.22 ± 0.60
MaxLL	5.19 ± 0.80	4.65 ± 0.97
AveLL	3.80 ± 0.65	3.12 ± 0.85

The results of Levene’s test and Student’s *t*-test of LCF are provided in Table 2, from which we can discover unequal variance in variable MaxLL and AveLL, but not in variable MaxUL and Ave UL. We adopted the adjusted data according to the Levene’s test. Both the maximum and average lower LCFs in the subject group (Group 1) was significantly different from those of the patients in the control group (Group 2, P < 0.001). The upper LCF showed no discernible differences between the two groups (P > 0.05).

**Table 2:** **Levene’s test and student’s *t*-test of LCF**

	**Levene’s test**		***t*-test for equality of means**						
	**F**	**Sig.**	**t**	**df**	**Sig. (2-tailed)**	**Mean difference**	**Std. error difference**	**95% confidence interval of the difference**	
								**Lower**	**Upper**
MaxUL	17.233	.000	-.282	131	.778	-.041	.146	-.331	.248
AveLL	35.573	.000	-1.677	131	.096	-.281	.167	-.612	.050
MaxLL	1.196	.276	3.513	130.674	.001	.541	.154	.236	.845
AveLL	2.853	.094	5.204	128.774	.000	.681	.131	.422	.940

Max UL indicates maximum upper lip closing force; Ave UL, average upper lip closing force; Max LL, maximum lower lip closing force; Ave LL, average lower lip closing force;

The *t*-test result of MaxLL and AveLL were adjusted according to the Levene’s Test.

In the correlation analyses (Table 3), the IMPA angle showed a high level of negative correlation with both the maximum and average lower LCFs of the patients in the subject group (P < 0.001). The IMPA produced r values of -0.789 (vs. MaxLL) and -0.697 (vs. AveLL), respectively. The FMA angle showed a significant positive relationship with the lower LCFs of patients with mandibular prognathism (P < 0.001) and produced r values of 0.672 (vs. MaxLL) and 0.582 (vs. AveLL), respectively.

**Table 3:** Results of pearson correlations

		**MaxUL**	**AVeUL**	**MaxLL**	**AveLL**
SNA	r	-.121	.039	-.223	-.174
	Sig.	.349	.764	.082	.175
SNB	r	.002	.126	-.177	-.095
	Sig.	.987	.328	.168	.463
ANB	r	-.195	-.108	-.116	-.150
	Sig.	.128	.405	.371	.245
pp-FH	r	.003	.040	-.043	-.049
	Sig.	.980	.757	.740	.705
op-FH	r	-.217	-.064	-.078	.013
	Sig.	.091	.622	.544	.922
overbite	r	.033	.028	-.425^**^	-.255^*^
	Sig.	.797	.831	.001	.045
overjet	r	-.144	-.072	-.337^**^	-.131
	Sig.	.265	.577	.007	.312
U1-FH	r	-.134	-.090	.032	.043
	Sig.	.299	.486	.808	.740
FMA	r	.001	-.003	.672^**^	.582^**^
	Sig.	.995	.981	.000	.000
IMPA	r	.110	.083	-.789^**^	-.697^**^
	Sig.	.396	.523	.000	.000
Ans_Ptm	r	-.003	-.094	.220	.065
	Sig.	.980	.466	.086	.615

**. Correlation is significant at the 0.01 level.

*. Correlation is significant at the 0.05 level.

**Figure 4:**
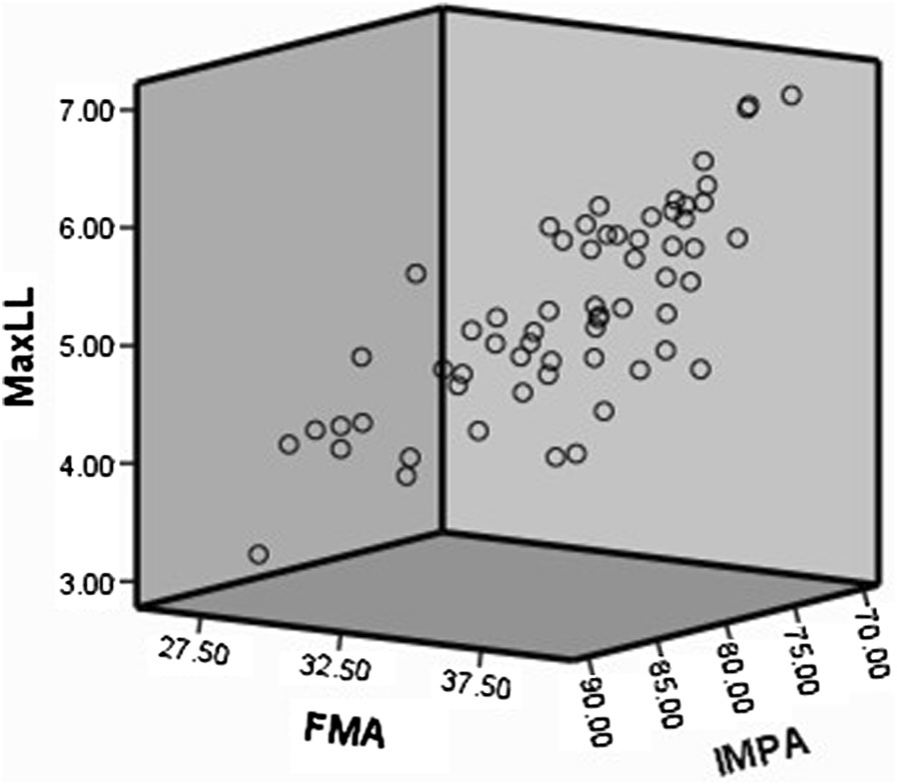
Linear relationship MaxLL, FMA and IMPA.

**Figure 5:**
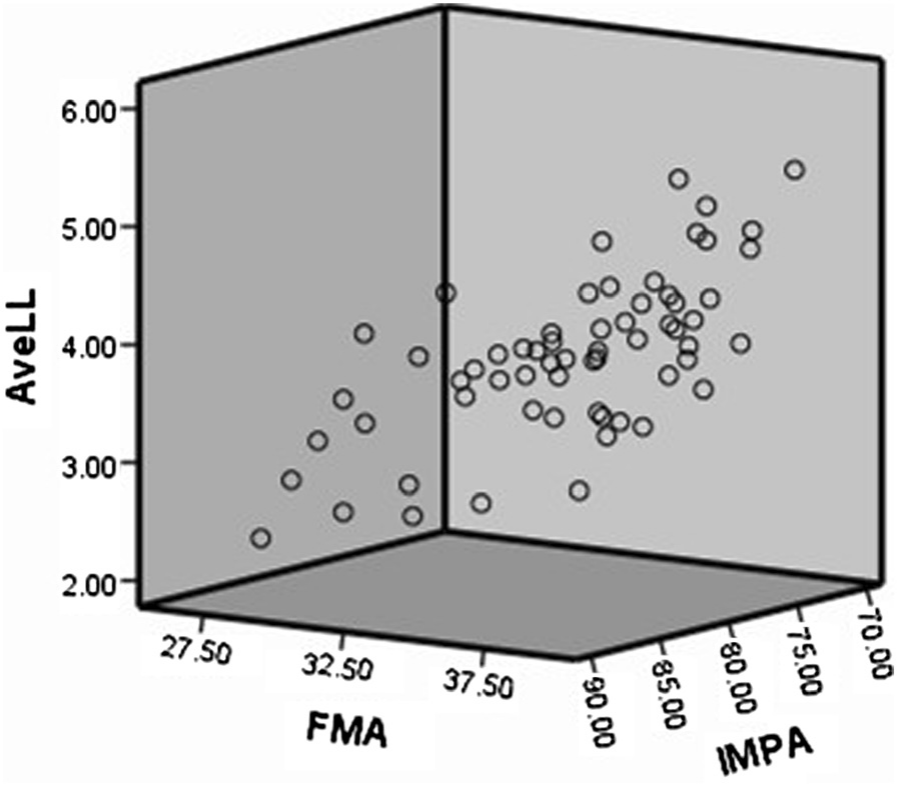
Linear relationship AveLL, FMA and IMPA.

The result of stepwise multiple linear regression analysis is presented in Table 4. 3-D scatter plots are showed in Figure 4 and Figure 5. With regard to the results of stepwise regression analysis, the maximum lower LCF was determined by an equation using the IMPA and FMA as follows: (MaxLL) = 12.192 - 0.125 * (IMPA) + 0.082 (FMA). The average lower LCF was determined by an equation using the IMPA and FMA as follows: (AveLL) = 9.112 - 0.091 * (IMPA) + 0.054 (FMA).

**Table 4:** Stepwise multiple linear regression analysis

**Dependent variable**		**Unstandardized coefficients**		**Standardized coefficients**	**t**	**Sig.**
		**B**	**Std. error**	**Beta**		
MaxLL	(Constant)	12.192	2.119		5.753	.000
	IMPA	-.125	.019	-.602	-6.538	.000
	FMA	.082	.024	.308	3.348	.001
AveLL	(Constant)	9.112	2.096		4.347	.000
	IMPA	-.091	.019	-.544	-4.840	.000
	FMA	.054	.024	.253	2.253	.028

## Discussion

Phenotypic heterogeneity and variation in clinical severity typify the diversity found within the complex class of occlusal morphologies grouped under the umbrella term of “Class III malocclusion” [18]. Definitions of the condition have varied throughout the orthodontic, dental, anatomical, and anthropological literature. Mackay [4] identified five Class III subgroups, all of which exhibited mandibular prognathism. Because of the high prevalence of mandibular prognathism in Class III patients, we chose this group of patients as our target subjects. A greater degree of lip pressure was noted in men than in women in previous studies [16,19], so all the subjects in our study were women to eliminate this type of variation.

Etiologic constituents of Class III malocclusion have turned out to be quite complex. It might appear that the variations in dental arch morphology, tooth position and skeletal components could account for Class III relationships; also, the contribution of the soft tissue matrices cannot be overlooked. It is conceivable that Class III malocclusions might result from the activity of the circumoral musculature, to some degree. For example, hypofunction of mentalis muscle appears to be related to mandibular protrusion [8]. Bardach [20] et al. tested the hypothesis that cleft lip repair contributed to maxillofacial growth aberrations. They found that undermining the soft tissue of the upper lip on the surface of the maxilla was detrimental to maxillofacial growth in beagles.

Teeth encounter even bigger force during oral functions, such as chewing, speaking and swallowing. The impact of perioral force under functional circumstance is rather significant, but it is technically difficult to measure it. Large interindividual variation is a common finding in studies of muscle pressure [21,22]. It would be very difficult to control the interindividual variation because of the varied functional response among individuals. Bundgaard [23] stated that similarities in occlusion and facial morphology do not account for similarities in the functional pattern. Due to the variation of functional position status, we chose peak passive lip pressure to represent it. It is also undeniable that most of the time lips are at rest, so that the static pressure for the lips also generates remarkable influence on the teeth and jaws.

Preceding studies [9-16] have shown higher upper LCF in Class I malocclusion. Among our subjects, both the average and maximum upper LCF of Class I patients showed no differences with those of the patients in the subject group (Table 2, P > 0.05). The different result possibly arose from distinct eligibilities of experimental design. According to Ruan’s [24] theory, decreased upper lip pressure was due to maxillary retrognathia, which is one of etiologies of Class III malocclusion. However, many factors may lead to Angle Class III condition, including maxillary hypodevelopment, mandibular overdevelopment or a combination of these two. To remove the confounding factors, the subjects in our study were of normal maxillary development (average SNA 80.96 ± 1.37, within the normal range), so it may be explicable that the upper LCF showed no difference with that of control group in our study.

Lip force pattern has been demonstrated to be related to skeletal dysplasia in the maxillofacial region [9,11-15]. Thüer and Ingervall [15] measured the lip pressure of children with varying types of malocclusions and indicated that the lip pressure exerted on the upper incisors was higher in Class II division 1 than in Class I malocclusions and was lowest in children with Class II division 2 malocclusions. In our study, both average and maximum lower LCFs of the patients with mandibular prognathism was considerably higher than those of the control group (Table 2, P < 0.001). In terms of biomechanics, the lower lip closing force was generated by the contraction of orbicularis oris muscle and mentalis muscle. The orbicularis oris muscle, annular and flat, is located in the lips around the rima oris. The mentalis muscle makes its insertion from the mandible and they interlace the orbicularis oris to an extensive degree. Overdevelopment of the mandible in vertical direction resulted in downwards and backwards rotation, which may initiate the growth remodelling of these two muscles by their contraction direction and pressure tension.

Most authors have accepted the equilibrium theory of tooth position [13,25]. The tongue, cheeks, and lips are the most critical environmental determinants of tooth position. LCF plays a significant role in guiding tooth eruption and in maintaining dental arch formation and stability. In our research, the IMPA angle was significantly negatively correlated with the lower LCF (Table 3, P < 0.001). IMPA, standing for the lower incisor-mandibular plane angle, reflects the relative inclination of the lower incisor towards the mandible. Different from the normal condition, the overdevelopment of the mandible positioned the lower incisors in front of the upper incisors. This kind of situation will make the incisors lose their normal function of biting and cutting. The compensatory mechanism thus arose to make the lower incisors leaning in the lingual direction to match the upper teeth, so that the patients can eat normally.

The ANB angle usually serves as an eminent representative of the skeletal pattern, while the SNB angle has commonly been used to describe the mandibular anteroposterior relationship to the cranial base. In our research, LCF showed no discernible relationship with these skeletal indicators (Table 3, P > 0.05), indicating that LCF might have little relationship with the formation of the skeletal pattern. More likely, compared to lip force, heredity plays a more substantial role in contributing to the skeletal pattern development of MP [26] through gene function [27,28] or other neuromuscular factors.

Class III patients with long faces are widespread in Asia [2,29]. In our research, the FMA angle was substantially correlated with lower LCF (Table 3, P < 0.001). FMA, standing for the Frankfort Horizontal-Mandibular Plane Angle, reflects vertical relation of facial hard tissue and development direction of mandible. With the FMA getting larger, followed by an increase in the anterior face height, the mandible rotated downwards and backwards. That change may influence the direction and tension of perioral muscles. However, some studies have indicated that patients with low mandibular plane angles have greater development of their perioral and masticatory musculature than those with high angles [30,31]. Their results contained the development of masticatory musculature, including masseter, medial, lateral pterygoid muscle, etc. However, in our research, the object was restricted to the lip closing force, which is mainly generated by the orbicular muscle and the mentalis. In addition to this, the data varies significantly among different races and ethnicities, and also different test instrument could make a contribution to the discrepancy.

It was reasonable that causal relationships were more critical than correlations in explaining disease mechanism. In our research, we found that lower lip closing force of mandibular prognathism patient was related to their craniofacial structure. Base on the existing data, we cannot conclude whether it was the skeletal deformity gave rise to the abnormal lip closing force or the opposite. The deep mechanism needs our further research design and practice. A longitudinal study may be engaged.

Variations in tissue growth and the development and function of force within the oral environment often result in different types of malocclusion [16]. A better understanding of the forces in tooth-adjacent areas could contribute to diagnosing, treating, and maintaining outcomes in orthodontic patients. Further research should be focused on the causal relationships and the means to intervene in dysfunctional lip forces in the early stages of different types of malocclusion, to reduce the difficulty of subsequent treatments. Further studies in molecular biology are also needed to identify gene-environment interactions so that we can undertake research to uncover the etiology of MP.

## Conclusions

The lower LCF of patients with mandibular prognathism was significantly higher than that of patients with Class I.

LCF had little impact on the formation of the jaws, but it had an influence on the position of incisors to a certain extent. The lower LCF was significantly negatively correlated with the IMPA.

The lower LCF was significantly positively correlated with the FMA.

## Abbreviations

MP: Mandibular prognathism; LCF: Lip closing force; FMA: Frankfort Mandibular Plane Angle; IMPA: L1 to mandibular plane angle.

## Competing interests

The authors declare that they have no competing interests.

## Authors’ contributions

SC participated in the design of the study and acquisition of data, and carried out the manuscript drafting. YC performed the statistical analysis and coordination and contributed to draft the manuscript. FC conceived of the study, and participated in its design. All authors read and approved the final manuscript.
